# Evolutionary functional elaboration of the *Elovl2/5* gene family in chordates

**DOI:** 10.1038/srep20510

**Published:** 2016-02-09

**Authors:** Óscar Monroig, Mónica Lopes-Marques, Juan C. Navarro, Francisco Hontoria, Raquel Ruivo, Miguel M. Santos, Byrappa Venkatesh, Douglas R. Tocher, L. Filipe C. Castro

**Affiliations:** 1 Institute of Aquaculture, School of Natural Sciences, University of Stirling, Stirling FK9 4LA, Scotland, UK; 2 CIIMAR – Interdisciplinary Centre of Marine and Environmental Research, U. Porto – University of Porto, Rua dos Bragas 289, 4050-123 Porto, Portugal; 3 ICBAS - Institute of Biomedical Sciences Abel Salazar, U. Porto - University of Porto, Rua de José Viterbo Ferreira 228, 4050-313 Porto, Portugal; 4 Instituto de Acuicultura Torre de la Sal (IATS-CSIC), Ribera de Cabanes 12595, Castellón, Spain; 5 Department of Biology, Faculty of Sciences, U. Porto - University of Porto, Rua do Campo Alegre, 4169-007 Porto, Portugal; 6 Institute of Molecular and Cell Biology, Agency for Science, Technology and Research, Biopolis, Singapore 138673

## Abstract

The biosynthesis of long-chain polyunsaturated fatty acids (LC-PUFA) provides an intriguing example on how multi-enzymatic cascades evolve. Essential LC-PUFA, such as arachidonic, eicosapentaenoic, and docosahexaenoic acids (DHA), can be acquired from the diet but are also endogenously retailored from C_18_ precursors through consecutive elongations and desaturations catalyzed, respectively, by fatty acyl elongase and desaturase enzymes. The molecular wiring of this enzymatic pathway defines the ability of a species to biosynthesize LC-PUFA. Exactly when and how in animal evolution a functional LC-PUFA pathway emerged is still elusive. Here we examine key components of the LC-PUFA cascade, the *Elovl2*/*Elovl5* elongases, from amphioxus, an invertebrate chordate, the sea lamprey, a representative of agnathans, and the elephant shark, a basal jawed vertebrate. We show that *Elovl2* and *Elovl5* emerged from genome duplications in vertebrate ancestry. The single *Elovl2/5* from amphioxus efficiently elongates C_18_ and C_20_ and, to a marked lesser extent, C_22_ LC-PUFA. Lamprey is incapable of elongating C_22_ substrates. The elephant shark *Elovl2* showed that the ability to efficiently elongate C_22_ PUFA and thus to synthesize DHA through the Sprecher pathway, emerged in the jawed vertebrate ancestor. Our findings illustrate how non-integrated “*metabolic islands*” evolve into fully wired pathways upon duplication and neofunctionalization.

The origin of complexity in living systems is a central question in evolution^1^,^2^. Pairwise interactions between molecules (e.g. ligand and receptors; enzymes and their substrates) and the impact of gene duplication on protein function have provided crucial insight into the understanding of physiological diversity^3^. Additionally, the association of different enzymes into single pathways and how these are affected by evolutionary processes is fundamental to reconstruct the history of metabolic gene networks^4^,^5^. The biosynthesis of long-chain (C ≥ 20) polyunsaturated fatty acids (LC-PUFA) in animals represents a fascinating example, where phylogenetically unrelated enzymes participate in a metabolic cascade to synthesize vital molecules such as arachidonic acid (ARA, 20:4n-6), eicosapentaenoic acid (EPA, 20:5n-3) and docosahexaenoic acid (DHA, 22:6n-3)^6^,^7^ (Fig. 1A). In addition to dietary input, LC-PUFA are synthesized endogenously from essential C_18_ polyunsaturated fatty acid (PUFA) precursors including linoleic acid (LOA, 18:2n-6) and α-linolenic acid (ALA, 18:3n-3) in mammals and teleosts, through a series of consecutive desaturation and elongation reactions^8^ (Fig. 1A). How and when this gene pathway has emerged and functionally diversified over time is still obscure. Typically in mammals, the metabolic cascade converting C_18_ PUFA into bioactive C_20-22_ LC-PUFA, such as ARA, EPA and DHA requires the concerted action of distinct Δ5 and Δ6 fatty acyl desaturase (FADS) enzymes, as well as that of elongation of long-chain fatty acids (ELOVL) proteins including ELOVL2 and ELOVL5 at specific steps in the pathway^8^ (Fig. 1A). Recently, the ability for direct ∆4 desaturation of 22:5n-3 to 22:6n-3 has been also shown in human FADS2^9^. The mechanisms of LC-PUFA biosynthesis in teleost fish, particularly farmed species, have been extensively investigated in the past decades, and many aspects of these metabolic pathways are better understood in fish compared to mammals. For example, the specific ability to convert C_18_ PUFA into LC-PUFA is directly dependent on the exact *Fads* and *Elovl* gene repertoire as well as their substrate specificities^10^,^11^,^12^,^13^. It has been shown that the inability of most teleosts to utilize Δ5 desaturase substrates is linked to the specific loss of *Fads1*^11^,^12^. Surprisingly, the small number of teleost species able to perform Δ5 conversions have a *fads2* gene with Δ5 activity^10^,^14^,^15^,^16^.

Genes encoding ELOVL proteins have received comparatively less attention, although their action is critical for a complete and functional LC-PUFA pathway^17^ (Fig. 1A). Generally, mammalian ELOVL5 is involved in the elongation of C_18_ and C_20_ PUFA, whilst ELOVL2 is predominantly active towards C_20_ and C_22_ PUFA^18^,^19^ (Fig. 1A). In contrast, the bird ELOVL5 is, to some extent, able to convert docosapentaenoic acid (DPA, 22:5n-3) to C_24_ LC-PUFA, though with considerable less efficiency than ELOVL2, which displays a similar substrate preference to mammals^20^. The *elovl* gene repertoire in teleosts is also distinctive from that of tetrapods. Most species studied so far have a single *elovl5* gene with the ability to elongate C_18_ and C_20_ PUFA substrates, with marginal activity towards C_22_^21^,^22^,^23^,^24^,^25^, with Atlantic salmon appearing as the sole fish species where two copies of *elovl5* have been characterized^11^,^26^. In contrast, an *elovl2* orthologue has been identified only in Atlantic salmon^10^ (*Salmo salar*), zebrafish^27^ (*Danio rerio*) and rainbow trout^28^ (*Oncorhynchus mykiss*), and with ray-finned fishes (including most marine species) appearing to lack *elovl2* in their genomes^11^. Similar to their tetrapod counterparts, teleost *elovl2* demonstrated the capacity to elongate DPA and thus contribute to DHA production through the so-called “Sprecher pathway”^29^ (Fig. 1A). From the above, *Elovl5* appears to be unique in its capability to elongate C_18_ PUFA substrates and, similarly, *Elovl2* towards C_22_ PUFA, while there is an overlap between both enzymes in their capacity to metabolize C_20_ substrates. However, when exactly *Elovl2* and *Elovl5* genes diverged and their respective functional fatty acid preferences emerged in metazoan evolution is presently unknown. Interestingly, various mollusk species, including the common octopus (*Octopus vulgaris*), the noble scallop (*Chlamys nobilis*) and cuttlefish (*Sepia officinalis*), have been shown to possess an *Elovl* gene, phylogenetically basal to the vertebrate *Elovl2* and *Elovl5*^30^,^31^,^32^. Curiously, the mollusk Elovl enzyme is only capable of metabolizing C_18_ PUFA and to lesser extent C_20_^30^,^31^,^32^ (Fig. 1A). The desaturase abilities in mollusks are also markedly different when compared to mammals and teleosts, since only Δ5 desaturases have been described so far^33^,^34^,^35^ (Fig. 1A). These results suggest a complex scenario regarding the evolutionary emergence of a complete LC-PUFA biosynthetic pathway.

Despite the significant effort made to clarify the LC-PUFA biosynthetic capabilities in some vertebrate lineages, the presently known complement of *Fads* and *Elovl* genes and their biosynthetic abilities in key evolutionary lineages hampers the precise evolutionary profiling of this pathway. Here we investigate the *Elovl2*/*Elovl5* gene repertoire at a key evolutionary moment: the invertebrate/vertebrate transition (Fig. 1B). By examining three species, including the European amphioxus (*Branchiostoma lanceolatum*, cephalochordate), the sea lamprey (*Petromyzon marinus*, agnathan) and the elephant shark (*Callorhinchus milii*, basal gnathostome), we provide an insightful snapshot into the evolution of critical enzymes dictating the LC-PUFA biosynthetic pathways in chordates.

## Results

### Elovl2 and Elovl5 originated in the ancestor of vertebrates

**Figure 1:**
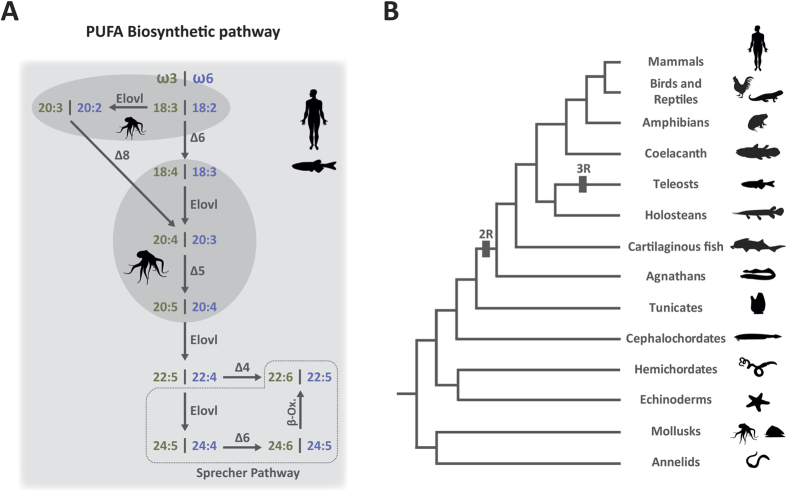
Biosynthetic pathway of LC-PUFA as determined in mammals and teleosts (all reactions shown), and octopus (confined to reactions in the two ellipses). (**A**). Elongation (Elovl), desaturation (Δ4, Δ5 and Δ6) and β-oxidation (β-oxi) reactions are indicated. The omega-3 (ω3) and omega-6 (ω6) PUFA synthesis cascades are shown in parallel. Each composite number (e.g. 18:3) refers to a specific PUFA, with the first number indicating the number of carbon atoms and the second referring to the ethylenic bonds (details on each PUFA in supplementary Table 1). Phylogenetic tree of the major Bilaterian animal groups considered in this study (**B**). Genome duplications are indicated (2R and 3R).

**Figure 2:**
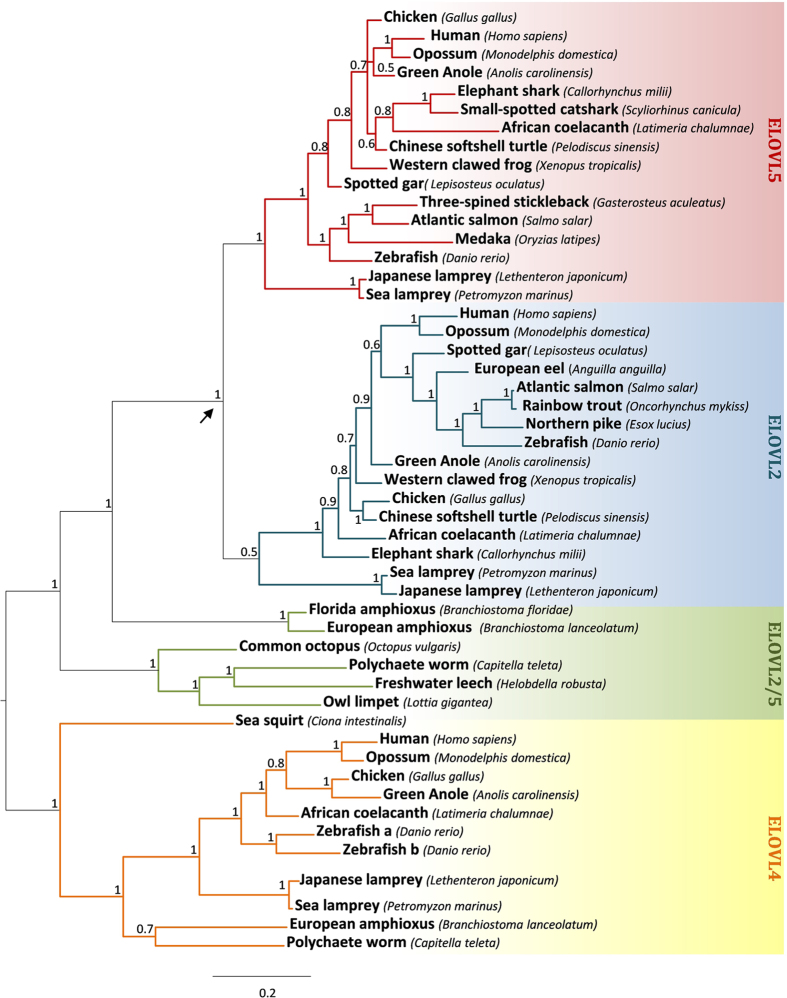
Bayesian molecular phylogenetic analysis of the *Elovl2, Elovl5 and Elovl4* genes. Numbers at nodes indicate posterior probabilities. Arrow denotes duplication timing of *Elovl2/5*. Rooted on the *Elovl4* clade. Accession numbers for all sequences are provided in the supplementary Table 2.

We analyzed the repertoire of *Elovl2* and *Elovl5* like genes in a total of 19 species representing all major vertebrate lineages (Sarcopterigii, Actinopterigii, Chondrichthyans and Agnathans) (Fig. 1B). In addition, we also investigated invertebrate species, representing four phyla from invertebrate protostomes and deuterostomes (Fig. 1B). The retrieved sequence dataset was used for phylogenetic reconstruction employing two methods, Bayesian analysis (BA) and Maximum likelihood (ML) (supplementary Fig. 1 for the ML phylogeny). We found two well-supported monophyletic groups, one containing all *Elovl4* sequences, and another containing invertebrate single copy *Elovl2/5* from cephalochordates and various protostome species and all vertebrate *Elovl2* and *Elovl5* sequences (Fig. 2). Within the latter group, gnathostome sequences formed two sister clades *Elovl2* and *Elovl5*, respectively. Each of the lamprey sequences branched together with gnathostome *Elovl2* and *Elovl5*, although with low statistical support in the case of *Elovl2* (Fig. 2). Therefore, the overall tree topology is indicative of the timing of *Elovl2/5* gene expansion, coincident with the evolution of the vertebrate lineage approximately 500 million years ago. No tunicate sequences were used in our analyses since no orthologues of *Elovl2/5* were found in genomes from sea squirts (*Ciona intestinatis* and *C. savignyi*) and the star ascidian (*Botryllus schlosseri*), despite the former having an *Elovl4*-like gene with the ability to elongate C_18_ and C_20_ PUFA^36^. Additionally, while some studies in teleosts have suggested that *Elovl4* can partly contribute to the LC-PUFA biosynthesis^37^ these enzymes are generally related to the biosynthesis of very long-chain (C > 24) fatty acids^38^, and thus were not considered in this study.

### Genome duplications generated Elovl2 and Elovl5 paralogues in vertebrates

**Figure 3:**
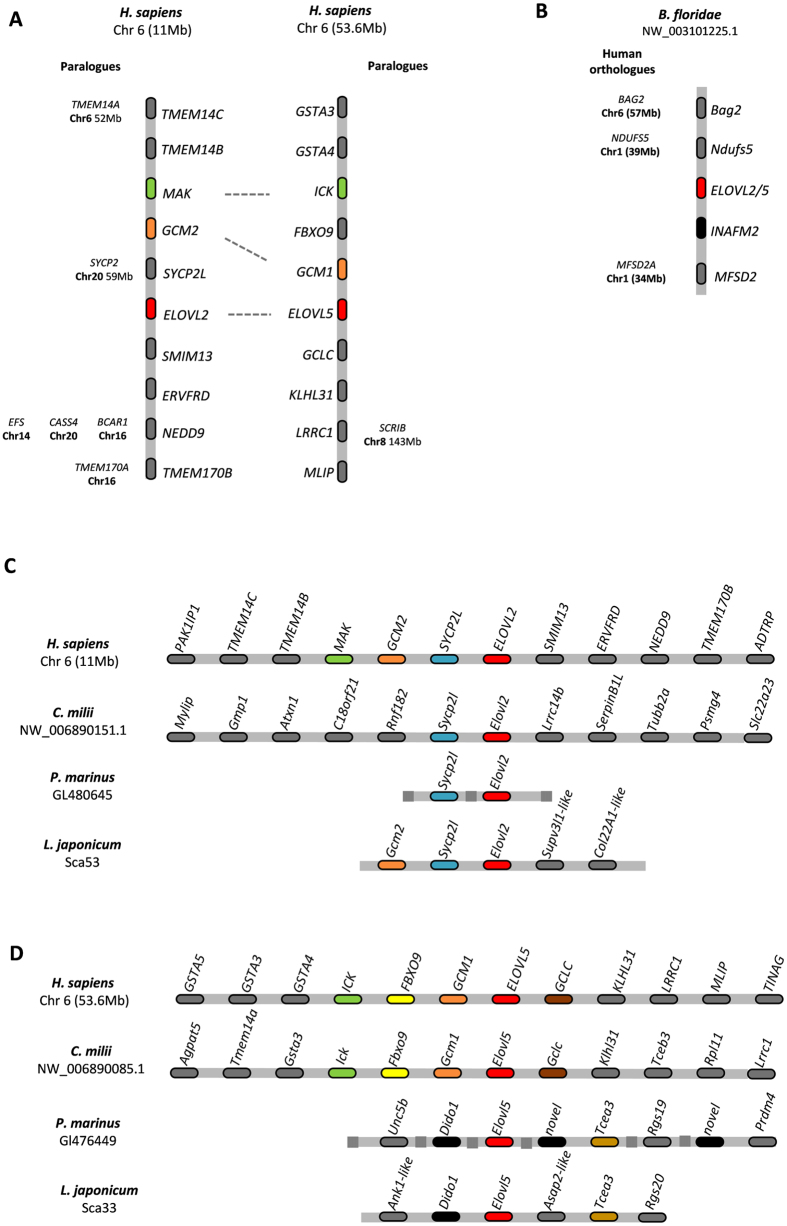
Comparative genomic maps of *Elovl* gene *loci*. (**A**) Paralogy analysis of *ELOVL2* and *ELOVL5* human orthologues; (**B**) the amphioxus *elovl2/5* gene *locus*; (**C**) synteny analysis of the *Elovl2* genes in lampreys, human and elephant shark; (**D**) synteny analysis of the *Elovl2* genes in lampreys, human and elephant shark.

The phylogenetic analysis supported the timing of *Elovl2* and *Elovl5* origin to the ancestor of vertebrates. Thus, we hypothesize that genome duplications were involved in the diversification of *Elovl2/5* genes. Human *Elovl2* and *Elovl5* localize to the same human chromosome (Hsa6) though at separate regions (Fig. 3A). These two genomic sections were linked to a four-fold paralogy originating from genome duplications^38^ (linkage group 4) involving a quartet of regions: paralogy A at Hsa20.5, paralogy B at Hsa2.1/6.6/6.8, paralogy C at Hsa6.2/8.2/8.4, and paralogy D at Hsa1.2 (supplementary Fig. 2). In effect, neighboring *Elovl2* and *Elovl5* gene families with a duplication history coincident with genome duplications, have, in most cases, a gene-by-gene paralogy in the expected regions (Fig. 3A). For example, *GCM1* and *ICK* (neighbors of *Elovl5*) have a vertebrate specific paralog mapping to the *Elovl2 locus*, *GCM2* and *MAK*, respectively. Also *Sycp2l*, localizing close to *Elovl2*, has a paralogue at Hsa20.5 as expected (paralogy group A) (Fig. 3A). Additionally, we also examined the *Elovl2/5* genomic *locus* of the pre-duplicated genome of the Florida amphioxus (*B. floridae*) (Fig. 3B). Coherently, we found that the neighboring genes *Bag2*, *Ndufs5* and *Mfsd2* have their human orthologues localizing to Hsa6 (close to *Elovl5*) and Hsa1, part of linkage group C and D, respectively (Fig. 3B, supplementary Fig. 2). Thus, we can conclude that *Elovl2* and *Elovl5* have appeared as part of whole-genome duplications.

### Are Agnathan Elovl genes exact Elovl2 and Elovl5 orthologues?

To further clarify the orthology of the identified *Elovl2/5* sequences, we examined the syntenic relationships of *Elovl2/5* genes in key species. Gnathostome *Elovl2* and *Elovl5* gene *loci* were conserved, though with different degrees (Fig. 3C, D; supplementary Fig. 3). For example, *Sycp2l* flanks *Elovl2* in humans and the elephant shark, indicative of a common origin (Fig. 3C). A strongly conserved syntenic pattern was also observed in the *Elovl5 locus*, with *Gcm1* and *Gclc* outflanking this gene in all gnathostome species except the former in zebrafish (Fig. 3D; supplementary Fig. 3). The exact orthology of agnathan gene sequences poses some challenges, namely when evolutionary processes such as whole genome duplications and gene loss are involved^40^,^41^,^42^. Given that the putative lamprey *Elovl2* was statistically weakly supported in the phylogenetic tree, we examined also the flanking gene families of the putative *Elovl* genes in both the sea lamprey and the Japanese lamprey (*Lethenteron japonicum*). In both species, the putative *Elovl2 locus* includes orthologues of *Sycp2l* and *Gcm2* gene, denoting a strong conservation with the human *locus* (Fig. 3C). In contrast, the “*Elovl5*” *locus* of lampreys displays no synteny conservation with other vertebrates (Fig. 3D). Although we cannot exclude that this represents a different paralogue retained uniquely in lampreys, we suggest that this is a *bona fide Elovl5* gene, in a highly rearranged *locus*.

### Functional characterization of amphioxus, sea lamprey and elephant shark ELOVL enzymes

We next analyzed the substrate specificities of ELOVL enzymes from three chordate species, namely amphioxus, sea lamprey and elephant shark (Table 1). Transgenic yeast expressing the amphioxus *Elovl2/5* ORF were able to elongate C_18_, C_20_ and, to a lesser extent, C_22_ PUFA substrates (Table 1). The sea lamprey *Elovl5* showed relatively high activity towards C_18_ PUFA (18:4n-3 and 18:3n-6), and lower activity toward the C_20_ PUFA (20:5n-3 and 20:4n-6). Compared to the sea lamprey *Elovl5*, the *Elovl2* was very efficient in the elongation of C_20_ to C_22_, with C_18_ PUFA being elongated to a lesser extent (Table 1). Interestingly, neither of the sea lamprey *Elovl* enzymes displayed the capacity to elongate C_22_ to C_24_ (Table 1). In order to investigate when the *Elovl2* acquired the ability to elongate C_22_ PUFA, we tested the function of the elephant shark *Elovl2*. Consistent with the activities exhibited by fish and mammalian orthologues^16^,^43^ the elephant shark *Elovl2* had marginal activity towards C_18_ PUFA and high elongation capability on C_20_ and C_22_ PUFA that were converted into the corresponding C_22_ and C_24_ elongation products, respectively (Table 1). Moreover, the functional characterization of the elephant shark *Elovl5* confirmed its ability to elongate preferably C_18_ and C_20_ to C_20_ and C_22_ PUFA (Table 1), respectively, as typically observed in other vertebrate lineages^16^,^17^.

### W231C substitution confers C_22_ to C_24_ elongation capacity to sea lamprey Elovl2

Functional characterization of the sea lamprey *Elovl2* showed no ability to elongate C_22_ PUFA to C_24_ products contrary to those of gnathostome *Elovl2*. On the other hand, elephant shark *Elovl2*, whose sequence contains the specific cysteine (C) residue regarded as critical for elongation of C_22_ by *Elovl2*^41^ (supplementary Fig. 4), showed ability to elongate C_22_ PUFA as in gnathostome lineages. Coherently, the sea lamprey *Elovl2* exhibits a tryptophan (W) typical of *Elovl5* sequences (supplementary Fig. 4). Thus, we next tested whether site-directed mutagenesis of W231C would drift the enzymatic activity towards C_22_ PUFA elongation as observed in the gnathostome orthologue. Our mutagenesis analysis showed that the W231C substitution conferred the sea lamprey *Elovl2* the ability to elongate 22:5n-3 to 24:5n-3, although the conversion obtained in the yeast expression system (2%) was notably lower when compared to other *Elovl2* proteins characterized in the present study and previously reported using similar systems^11^,^27^ (Table 1). Interestingly, the mutant retained its ability to elongate C_20_ PUFA such as 20:5n-3 and 20:4n-6 to the corresponding C_22_ PUFA, 22:5n-3 and 22:4n-6, but lost its ability to elongate C_18_ PUFA (Table 1). Overall, the functional characterization of sea lamprey *Elovl2* mutant confirms that the cysteine (C) residue indicated above is key for the C_22_ to C_24_ elongation ability^42^, but the relatively low conversion observed in the yeast system suggests that other amino acids are also critical for an efficient conversion of C_22_ into C_24_ PUFA.

## Discussion

Vertebrate radiation encompassed the acquisition of key physiological and anatomical innovations, as a consequence of gene and genome duplications^44^,^45^,^46^,^47^. Among others, these might have facilitated the challenge of colonizing new ecological niches with diverse nutrient composition such as, for example, LC-PUFA. ELOVL are key enzymes involved in the rate-limiting step of fatty acid elongation pathway by which β-ketoacyl-CoA is produced after the condensation of acyl-CoA molecule and malonyl-CoA^17^. Although these enzymes have been extensively studied in a number of metazoans including invertebrates and vertebrates, their evolution has yet to be deciphered. Here we focused on a subset of *Elovl* genes, namely *Elovl2* and *Elovl5*, critical in the biosynthetic pathways of LC-PUFA^17^. Combining phylogenetics, comparative genomics and functional data, we have been able to deduce the early evolution of functional *Elovl* specificities in chordates.

**Figure 4:**
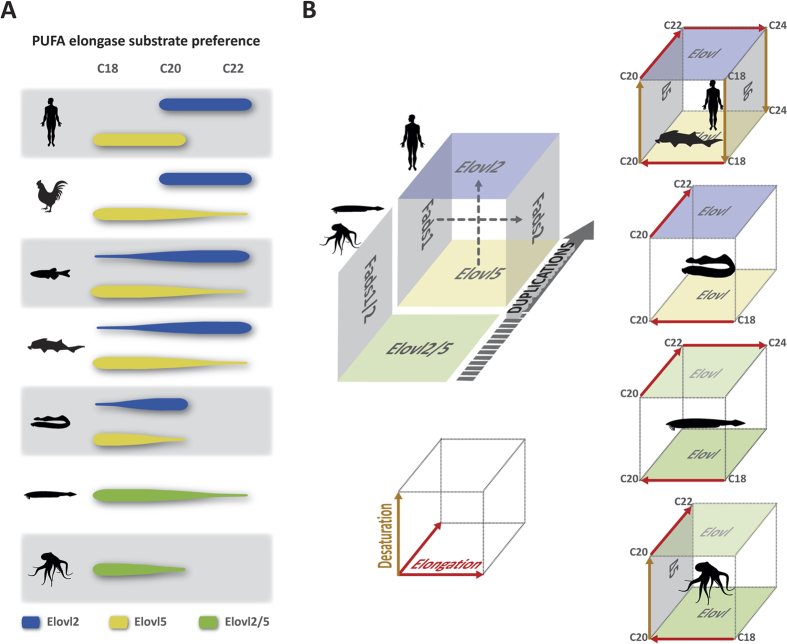
Evolutionary scenario of LC-PUFA biosynthesis functional diversification in Bilateria. (**A**) PUFA elongase substrate preference in octopus, amphioxus, lamprey, elephant shark, zebrafish, chicken and human; (**B**) schematic view of gene duplication events in the elongation/desaturation network along the invertebrate/vertebrate transition (top left) and known enzymatic activities of FADS and ELOVL (right; from top to bottom: octopus, amphioxus, lamprey and the gnathostomes elephant shark and human); Δ5 and Δ6 denote desaturation activities.

Phylogenetics and synteny revealed that orthologs of *Elovl2* and *Elovl5* occur only in vertebrate species. Thus, our data support that both *Elovl5* and *Elovl2* have evolved in agnathans, chondrichthyans, holosteans (spotted gar) and teleosts such as zebrafish and Atlantic salmon. Contrarily to *Elovl5*, *Elovl2* is absent in most of the ray-finned fish branch due to a gene loss event as previously hypothesized^11^. Moreover, the finding of a single *Elovl2/5* sequence in invertebrate deuterostomes and protostomes, and its basal position in the tree, defines the transition from invertebrate chordates to vertebrates as the exact timing at which diversification of *Elovl2/5* gene family occurred. Typically in mammals, ELOVL2 are enzymes with high elongation efficiency towards C_20_ and C_22_ PUFA, and marginal (if any) activity towards C_18_ substrates^8^,^17^ (Fig. 4A). In contrast, ELOVL5 have C_18_ and C_20_ PUFA as preferred substrates, but have little or no capability to elongate C_22_ PUFA^8^,^17^. The former elongation specificity is largely exhibited by protostomes such as the octopus *Elovl2/5*^30^ (Fig. 4A). In agreement, the here reported amphioxus ELOVL2/5 enzyme showed the same elongation pattern with C_18_ and C_20_ PUFA appearing as preferred substrates for elongation, although some ability to elongate C_22_ PUFA was also observed (Fig. 4A). The substrate preferences of the sea lamprey *Elovl2* and the *Elovl5* enzymes showed a complete inability to elongate C_22_ PUFA, whereas the elephant shark *Elovl2* was able to effectively elongate C_22_ PUFA, 22:5n-3 and 22:4n-6, to their corresponding C_24_ products as shown in teleosts and mammalian ELOVL2 proteins^11^,^17^,^27^,^28^ (Fig. 4A). The amino acid alignment of the various *Elovl2/5* sequences allowed us to identify that the elephant shark *Elovl2*, similar to orthologues from other gnathostome lineages including mammals, birds, amphibians and teleosts, contained within its sequence the cysteine (C) regarded as critical for C_22_ PUFA elongation by *Elovl2*^43^, while this residue was substituted by a tryptophan (W) in the sea lamprey *Elovl2*. Using a site-directed mutagenesis approach, we showed that the mutated lamprey *Elovl2* protein lost the ability to elongate C_18_ PUFA exhibited by the native protein and, more importantly, gained the ability to elongate C_22_ PUFA. However, the minute capacity to elongate C_22_ exhibited by the mutated lamprey *Elovl2* suggests that other unidentified amino acids are also critical for this function.

Apart from elongase activity, the complexity of the LC-PUFA biosynthetic network cannot be dissociated from LC-PUFA desaturation profiles. The combined analysis of *Fads* and *Elovl* gene repertoire and function in various species allows us to propose that a fragmented LC-PUFA pathway existed early in evolution (Fig. 4B). Data derived from mollusks strongly suggests that the ancestral bilaterian LC-PUFA biosynthetic pathway was composed of *Fads* and *Elovl* genes encoding, respectively, proteins with single desaturation (Δ5) and elongation (C_18_ to C_22_) enzymatic abilities^30^,^31^,^32^,^33^,^34^,^35^ (Fig. 4B), although the presence of additional uncharacterized desaturases in mollusks impedes a final conclusion^48^. An incomplete pathway also appears to exist in cephalochordates. Despite the functionalities of *Elovl2/5* showing its ability to elongate PUFA ranging from C_18_ to C_22_, a full complement of desaturase abilities is likely absent as suggested by *in silico* searches, with a single Fads-like gene described so far in their genome^12^ (Fig. 4B). However, relevant levels of DHA were found in the digestive tract of amphioxus^49^. While they could be exclusively diet-derived, an endogenous DHA production cannot be excluded. Thus, the characterization of the single amphioxus FADS should be addressed in the future. In agnathans, on the other hand, the restricted elongation profiles demonstrated by the lack of elongation activity by both Elovl-like enzymes towards C_22_ may limit the LC-PUFA biosynthetic pathways regardless of the possible number of genes or desaturase activities existing in lampreys (Fig. 4B). Importantly, the combined activities of the elephant shark *Elovl5* and *Elovl2* enabling elongation up to C_24_ LC-PUFA and thus DHA biosynthesis^29^, as well as the existence of Δ6 and Δ5 Fads in chondrichthyans^12^, strongly suggest that a fully developed LC-PUFA biosynthetic pathway dependent on the sequential action of *Elovl* and *Fads* was first operational in gnathostomes (Fig. 4B). The overall pathway has been conserved throughout this lineage with localized episodes of gene loss, gene duplication and functional plasticity as demonstrated by the Δ5 capacity of some teleost *Fads2*^12^.

However, it is difficult to foresee the exact evolutionary drivers accounting for the acquisition of a full biosynthetic pathway for LC- PUFA in organisms that have a likely supply in the diet. Clearly though, endogenous production of DHA, the final LC-PUFA in the cascade, is physiologically advantageous since it represents an additional source to cope with potential dietary scarcity, as well as satisfy particularly high requirements in early development^50^. Additionally, DHA levels are known to be especially high in tissues such as brain and retina, in mammals, teleosts and chondrichthyans^27^,^50^,^51^. Thus, it is conceivable to hypothesize that the elaboration of brain and eye function in vertebrate ancestry^52^, was paralleled by the capacity to endogenously regulate and synthesize DHA independently of exogenous sources.

In conclusion, the observed lineage-specific LC-PUFA biosynthetic profiles in chordate species were tailored by gene duplication events followed by enzymatic neofunctionalizations. We propose that the biosynthesis of the essential fatty acid DHA through the Sprecher pathway from C_18_ precursors was not fully resolved until gnathostomes emerged.

## Methods

### Sequence collection

ELOVL amino acid (aa) sequences were retrieved from Ensembl, GenBank, JGI (Joint Genome Institute), elephant shark genome project (http://esharkgenome.imcb.a-star.edu.sg/) and Japanese lamprey genome project (http://jlampreygenome.imcb.a-star.edu.sg/), databases through Blastp searches using as reference the annotated human ELOVL2, ELOVL5 and ELOVL4 aa sequences. Accession numbers are available in supplementary Table 2.

### Phylogenetic analysis

A total of 50 ELOVL aa sequences were aligned with MAFFT^53^ (L-INS-i method). The sequence alignment was stripped from all columns containing gaps leaving 200 gap free sites for phylogenetic analysis. Bayesian phylogenetic analysis was performed using MrBayes v3.2.3 available in CIPRES Science Gateway V3.3^54^. MrBayes was run for 5 million generations with the following parameters: rate matrix for aa = mixed, nruns = 2, nchains = 4, temp = 0.2, sampling set to 500 and burin to 0.25. Maximum likelihood phylogenetic analysis was performed in PhyML v3.0 server^55^ protein evolutionary model was calculated in PhyML using smart model selection resulting in JTT +G6 +I +F and the number of bootstrap replicates was set to 1000. The resulting trees were visualized in Fig Tree V1.3.1 available at http://tree.bio.ed.ac.uk/software/figtree/ and rooted with ELOVL4 sequences.

### Synteny and comparative genomics

*Elovl*2 and *Elovl*5 genes were mapped onto the respective species genomes, using the latest genome assemblies available in Ensembl release (Ensembl release 80, May 2015). The elephant shark genomic information was collected from Ensembl Pre assembly ESHARK1 (http://ensembl.fugu-sg.org/index.html) and for Japanese lamprey synteny maps were inferred using the draft assembly LetJap1.0 available at GenBank. When possible, we analyzed a 1Mb window centered on the corresponding *Elovl* gene, using the human *locus* as reference for comparison. Paralogy studies used the ancestral chordate genome reconstruction^39^. Ensembl paralog and ortholog prediction tools were used to infer evolutionary history of flanking *Elovl* genes in addition to phylogenetic analysis reconfirmation using ML methods.

### Elovl full ORF genes in amphioxus, sea lamprey and elephant shark

Total RNA was isolated from amphioxus (whole animal) and sea lamprey (kidney, liver and brain) using an Illustra RNAspin Mini RNA Isolation Kit (GE Healthcare, UK). All steps were performed according to the manufacturer’s recommendations, including the on-column treatment of isolated RNA with RNase-free DNaseI. One μg RNA was used for cDNA synthesis using the iScript cDNA Synthesis Kit (Bio-Rad) and following the manufacturer’s specifications. Initial isolation of the *Elovl-like* gene in amphioxus was achieved by PCR with Phusion® Flash (high-fidelity PCR master mix) using degenerate primers (supplementary Table 3). Initial PCR product was confirmed by sequencing and used to design gene specific primers (GSP) used obtain the full-length cDNA sequences by RACE PCR (SMARTer™ RACE cDNA Amplification, Clontech). For the sea lamprey, one complete and one incomplete *Elovl2/5*-*like* sequences were identified in the available genome. To obtain the open reading frames (ORF) of the incomplete *Elovl2/5*-*like* sequence (ENSPMAG00000005149), we carried out a RACE PCR. The elephant shark *Elovl2* sequence was identified in the transcriptome and genome sequence^56^, and was chemically synthesized (Integrated DNA Technologies, Inc., Glasgow, UK).

### Cloning into pYES2 vector and functional assays in yeast

Functional characterization of the ELOVL gene products from amphioxus, sea lamprey and elephant shark were investigated by heterologous expression in yeast *Saccharomyces cerevisiae* (strain InvSc1, Invitrogen). Briefly, the ORF of the target genes were cloned into the yeast expression vector pYES2 (Invitrogen) following a two-step routine. First, PCRs with specific primers flanking the full ORF were designed in the 5′ and 3′ UTR of each gene (supplementary Table 3) were performed using Phusion® Flash (high-fidelity PCR master mix) under the following conditions: initial denaturation at 98 °C for 10 s, followed by 25 cycles at 98 °C for 1 s annealing for 5 s and 72 °C for the required amount of time according to the product size. The second step consisted in re-amplification of the initial PCR product (diluted 1/50) with a set of primers containing the start and stop codons and restriction enzyme sites for further cloning into pYES2 (supplementary Table 3). PCR conditions were the same with the exception of the number of cycles that was increased to 35. The resulting PCR product was purified, digested with appropriate restriction enzymes and ligated into a similarly restricted pYES2 vector to produce the constructs pYES2-BlELOVL for *B. lanceolatum Elovl2/5*, pYES2-PmELOVL2 and pYES2-PmELOVL5 for *P. marinus Elovl2* and *Elovl5*, respectively, and pYES2-CmELOVL2 and pYES2-CmELOVL5 for *C. milii Elovl2* and *Elovl5*, respectively. Lamprey *Elovl2* W231C mutant was produced by site directed mutagenesis PCR using pYES2-PmELOVL2 as template, and the PCR product was subsequently purified, digested with the restriction enzymes and ligated into pYES2 to produce pYES2-PmELOVL2-W231C. Accuracy of the DNA sequences was confirmed in all constructs by sequencing. Transformation and culture of yeast were conducted as previously described^10^,^21^. In order to assess the substrate specificity of the ELOVL enzymes from amphioxus, sea lamprey and elephant shark, transgenic yeast expressing the *Elovl* ORF were grown in the presence of the following PUFA substrates: 18:4n-3, 18:3n-6, 20:5n-3, 20:4n-6, 22:5n-3 and 22:4n-6. After 48 h of incubation, yeast were harvested, washed and total lipid extracted by homogenization in chloroform/methanol (2:1, v/v) containing 0.01% BHT^13^.

### Fatty acid analysis of yeast and elongation conversions

Fatty acyl methyl esters (FAME), prepared from total lipids extracted from harvested cells, were analyzed using a Thermo Gas Chromatograph (Thermo Trace GC Ultra, Thermo Electron Corporation, Waltham, MA, USA) fitted with an on-column injection system and a FID detector. Further confirmation of FAME was performed with an Agilent 6850 Gas Chromatograph system coupled to a 5975 series MSD (Agilent Technologies, Santa Clara, CA, USA). The elongation conversion efficiencies from exogenously added PUFA substrates were calculated by the proportion of substrate fatty acid converted to elongated products as (all product areas/(all product areas + substrate area)) x 100.

## Additional Information

**How to cite this article**: Monroig, Óscar. *et al*. Evolutionary functional elaboration of the *Elovl2/5* gene family in chordates. *Sci. Rep*. **6**, 20510; doi: 10.1038/srep20510 (2016).

## Supplementary Material

Supplementary Information

## Figures and Tables

**Table 1:** Functional characterization of the amphioxus *Elovl2/5*, the sea lamprey *Elovl5*, *Elovl2* and mutated *Elovl2*, and the elephant shark *Elovl5* and *Elovl2* in *Saccharomyces cerevisiae*.

FA substrate	FA product	*Amphioxus Elovl2/5*	*Sea lamprey Elovl5*	*Sea lamprey Elovl2*	*Sea lamprey*mutated *Elovl2*	*Elephant shark Elovl5*	*Elephant shark Elovl2*
18:4n-3	20:4n-3	21	56	9	0	69	7
18:3n-6	20:3n-6	55	40	0	0	74	3
20:5n-3	22:5n-3	87	12	88	57	65	85
20:4n-6	22:4n-6	88	8	25	8	56	82
22:5n-3	24:5n-3	14	0	0	2	5	43
22:4n-6	24:4n-6	4	0	0	0	2	37

Conversions were calculated according to the formula (all product areas/(all products areas + substrate area)) ×10.
