# Supplementary material for: Evolutionary functional elaboration of the Elovl2/5 gene family in chordates
Source: Sci Rep. 2016 Feb 9;6:20510. doi: 10.1038/srep20510 (PMC4746653; doi:10.1038/srep20510)
Supplement: Supplementary Information [file srep20510-s1.pdf]

Evolutionary functional elaboration of the *Elovl2/5* gene family in chordates

Óscar Monroig[#]*, Mónica Lopes-Marques[#], Juan C. Navarro, Francisco Hontoria, Raquel Ruivo, Miguel M. Santos, Byrappa Venkatesh, Douglas R. Tocher, L. Filipe C. Castro[*]

Supporting Information

Supplementary Figures

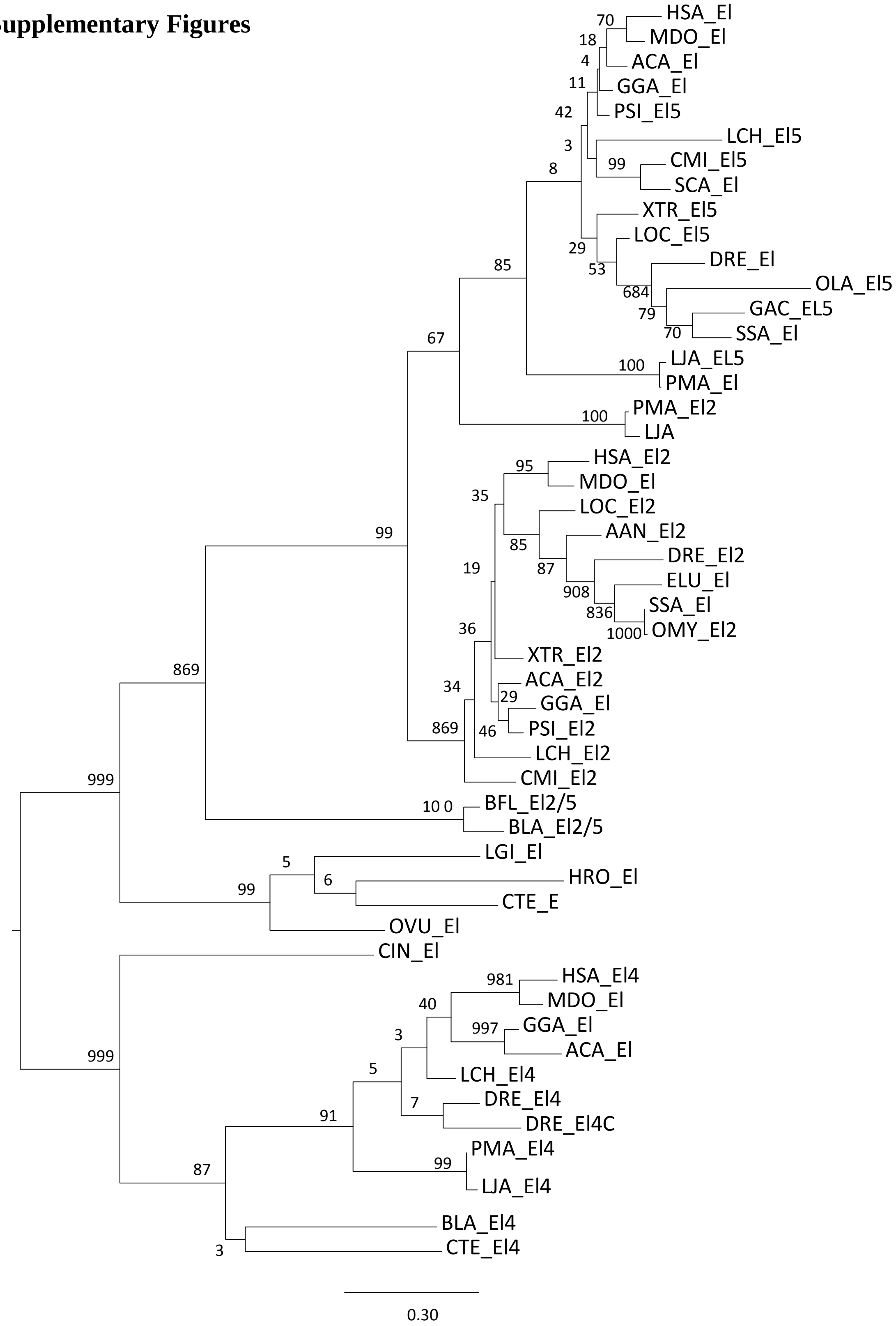

Figure 1. Maximum likelihood phylogenetic analysis was performed in PhyML v3.0 server (Guindon S et al 2010), protein evolutionary model was calculated in PhyML using smart model selection resulting in JTT +G6 +I +F and the number of bootstrap replicates was set to 1000. HSA – *H. sapiens*; MDO – *M. domestica*; ACA – *A. carolinensis* ; GGA – *G. gallus;* XTR – *X. tropicalis* ; PSI – *P. sinensis;* LCH – *L. chalumnae* ; DRE – *D. rerio;* GAC – *G. aculeatus* ; OMY – *O. mykiss*; OLA – *O. latipes*; SSA – *S. salar;* ELU – *E. lucius;* AAN – *A. anguilla;* CMI – *C. milii;* SCA – *S. canicula;* LJA – *L. japonicum;* PMA – *P. marinus;* LGI - *L. gigantea;* HRO – *H. robusta;* BFL – *B. floridae;* BLA – *B. lanceolatum;* CTE – *C. teleta;* OVU – *O. vulagris;* CIN – *C. intestinalis.*

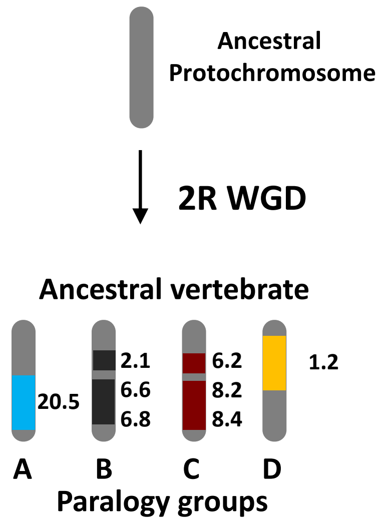

Figure 2. Distribution of the ancestral vertebrate paralogy groups containing *Elovl* genes in the human genome.

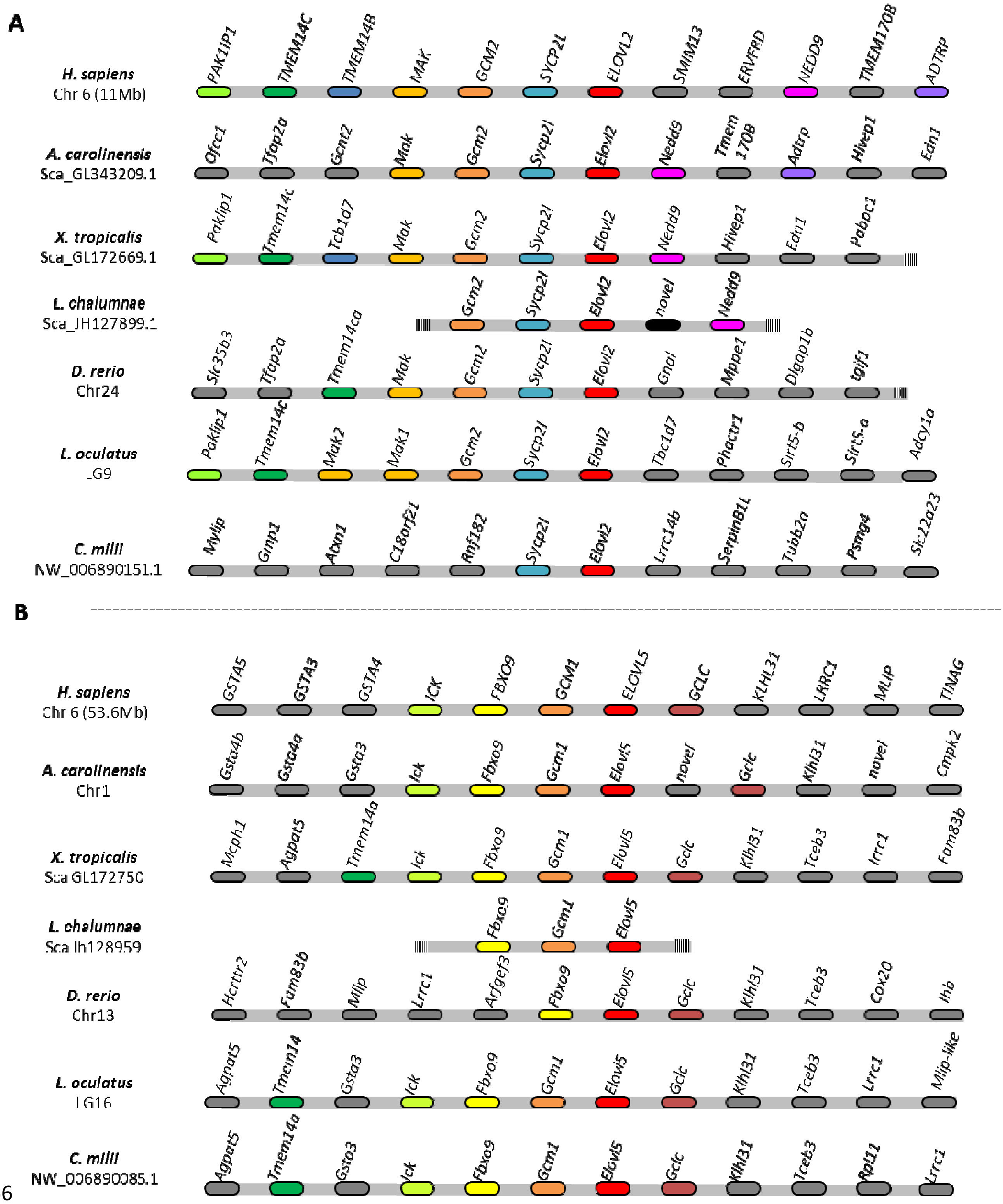

Figure 3. (A) Comparative synteny maps of the *Elovl2* locus and (B) *Elovl5* locus in *H. sapiens, A. carolinensis, X. tropicalis, L. chalumnae, D. rerio, L. oculatus* and *C. milii.*

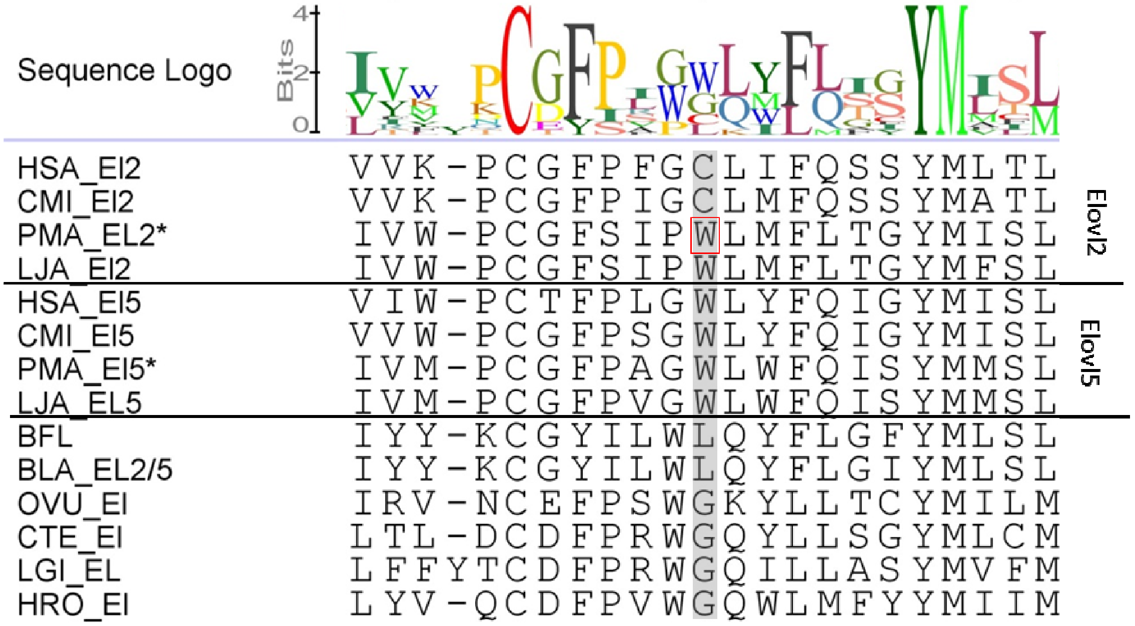

Figure 4. Sequence alignment identifying the mutated site in lamprey. HSA – *H. sapiens;* CMI – *C. milii,* PMA - *P. marinus,* LJA – *L. japonicum;* BFL – *B. floridae;* BLA – *B. lanceolatum ;* OVU – *O. vulgaris;* CTE – *C. teleta;* LGI – *L. gigantea;* HRO - *H. robusta.* *- denotes sequences isolated and cloned in this work, red box highlight mutation site W>C.

Supplementary Tables

Table 1. LC-PUFA in the biosynthetic cascade.

**ω3**	
**PUFA name**	**Symbol**
α-linolenic acid	18:3n-3
Stearidonic acid	18:4n-3
Eicosatetraenoic acid	20:4n-3
Eicosapentaenoic acid	20:5n-3
Docosapentaenoic acid	22:5n-3
Tetracosapentaenoic acid	24:5n-3
Tetracosahexaenoic acid	24:6n-3
Docosahexaenoic acid	22:6n-3
**ω6**	
**PUFA name**	**Symbol**
linoleic acid	18:2n-6
γ-linolenic acid	18:3n-6
Dihomo-γ-linolenic acid	20:3n-6
Arachidonic acid	20:4n-6
Adrenic acid	22:4n-6
Tetracosatetraenoic acid	24:4n-6
Tetracosapentaenoic acid	24:5n-6
Docosapentaenoic acid	22:5n-6

Table 2. Accession numbers of all sequences used in phylogenetic analysis.

<table>
<tr><th>Species</th><th>Elovl2</th><th>Elovl5</th><th>Elovl4</th></tr>
<tr><td>Human (Homo sapiens)</td><td>XP_011513019.1</td><td>NP_068586.1</td><td>NP_073563.1</td></tr>
<tr><td>Opossum (Monodelphis domestica)</td><td>XP_007488013</td><td>XP_001364339.1</td><td>XP_001366145.1</td></tr>
<tr><td>Chicken (Gallus gallus)</td><td>NP_001184237.1</td><td>NP_001186126.1</td><td>NP_001184238.1</td></tr>
<tr><td>Chinese softshell turtle (Pelodiscus sinensis)</td><td>XP_006138890</td><td>XP_006137802.1</td><td>-</td></tr>
<tr><td>Green Anole (Anolis carolinensis)</td><td>NP_001016159.1</td><td>XP_003215478.1</td><td>XP_003215742.1</td></tr>
<tr><td>Western clawed frog (Xenopus tropicalis)</td><td>NP_001016159.1</td><td>NP_001011248.1</td><td>-</td></tr>
<tr><td>African coelacanth (Latimeria chalumnae)</td><td>XP_006006450.1</td><td>XP_006010670.1</td><td>XP_006008610.1</td></tr>
<tr><td>Atlantic salmon (Salmo salar)</td><td>NP_001130025</td><td>NP_001117039.1</td><td>-</td></tr>
<tr><td>Rainbow trout (Oncorhynchus mykiss)</td><td>AIT56593.1</td><td>-</td><td>-</td></tr>
<tr><td>Northern pike (Esox lucius)</td><td>XP_010884057.1</td><td>-</td><td>-</td></tr>
<tr><td>Zebrafish (Danio rerio)</td><td>AAI29269.1</td><td>NP_956747.1</td><td>NP_957090.1
NP_956266.1</td></tr>
<tr><td>Medaka (Oryzias latipes)</td><td>-</td><td>XP_004077464.1</td><td>-</td></tr>
<tr><td>Three-spined stickleback (Gasterosteus aculeatus)</td><td>-</td><td>ENSGACT00000008538</td><td>-</td></tr>
<tr><td>Spotted gar (Lepisosteus oculatus)</td><td>XP_006634635.1</td><td>XP_006638754.1</td><td>-</td></tr>
<tr><td>European Eel (Anguilla anguilla)</td><td>JAH99109</td><td>-</td><td>-</td></tr>
<tr><td>Elephant shark (Callorhynchus milii)</td><td>XP_007900820/KT462565</td><td>XP_007892243.1/KT462566</td><td>-</td></tr>
<tr><td>Small-spotted catshark (Scyliorhinus canicula)</td><td>-</td><td>Transcript-ctg18611</td><td>-</td></tr>
<tr><td>Sea lamprey (Petromyzon marinus)</td><td>KT462563</td><td>KT462564</td><td>S4R5D2</td></tr>
<tr><td>Japanese lamprey (Lethenteron japonicum)</td><td>JL4990</td><td>JL3695</td><td>JL12276</td></tr>
<tr><td>Sea squirt (Ciona intestinalis)</td><td colspan="2">-</td><td>NP_001029014.1</td></tr>
<tr><td>Florida amphioxus (Branchiostoma floridae)</td><td colspan="2">JGI_211218</td><td>-</td></tr>
<tr><td>European amphioxus (Branchiostoma lanceolatum)</td><td colspan="2">KT462562</td><td>-</td></tr>
<tr><td>Common octopus (Octopus vulgaris)</td><td colspan="2">AFM93779.1</td><td>-</td></tr>
<tr><td>Polychaete worm (Capitella teleta)</td><td colspan="2">ELU18884.1</td><td>ELU05135.1</td></tr>
<tr><td>Owl limpet (Lottia gigantea)</td><td colspan="2">JGI protein Id 224291</td><td>-</td></tr>
<tr><td>Freshwater leech (Helobdella robusta)</td><td colspan="2">JGI protein Id 63042</td><td>-</td></tr>
</table>

Supplementary table 3. Details of all primers and PCR conditions.

Specie	Gene	Primer F	Primer R	Tm	Function
Branchiostoma lanceolatum	*Elovl2/5*	TGGTACTACTTCTCCAAGGCCathgarttyyt	TGGGCCTGGGTGATGTACykyttccacca	55	Degenerate primers
		CGCAGGATGAAGAACAACGTGTCA	GGCTAACTCGTTCATCCACGTCATC	65	GSP RACE primers
		TGCACTACCCACCATACGAA	TTTCAAATCGGTCGGATAGG	58	ORF
		CCCGGTACCAAGATGGCCACGACCACTGCAACTG	CCCTCTAGAGGTCATTCGGCTTTCTTAGCCCTCC	65	Cloning primers
Petromyzon marinus	*Elovl2*	-	CCGCCAGCCCGTAGTAGGAGTACAT	60	GSP RACE primers
		GGTATCAACGCAGAGTACATGG	AGTTTTGGACTAATCGCGTCAC	58	ORF
		CTTCTCCATCCCGTGTCTCATGTTCCTG	CAGGAACATGAGACACGGGATGGAGAAG	62	Site directed mutagenesis
		CCCGGTACCACCATGGAATTCTTGGATAACACACTCAATG	CCCTCTAGAGAATCGCCTCAGTCCAGAGCAACC	68	Cloning primers
	Elovl5	GCGCTTATGCTTACTGAATGT	TGGCATTTCCTTCTCTTCCAAT	58	ORF
		CCCGGATCCACAATGGAGGCACTGGACACAGC	CCCTCTAGATTACACGCGCTTGGGCTTGCGC	68	Cloning primers
